# Book Reviews

**Published:** 1906-03

**Authors:** 


### BOOK REVIEWS.

---

CULBRETH'S MATERIA MEDICA. A Manual of Materia Medica and Pharmacology for Students and Practitioners of Medicine and Pharmacy. Comprising all Organic and Inorganic Drugs which are and have been official in the United States Pharmacopœia, together with important Allied Species and Useful Synthetics. By David M. R. Culbreth, Ph. G., M.D., Professor of Botany, Materia Medica and Pharmacology in the University of Maryland, Departments of Medicine, Pharmacy and Dentistry. Fourth edition. Revised to accord with the new U. S. Pharmocopœia, 8th Decennial Revision. Octavo, 976 pages, 487 illustrations. Cloth, $4.75, net. Lea Brothers & Co., Publishers, Philadelphia and New York, 1906.

Since the appearance of this work, nine years ago, three editions have been exhausted. The present edition has been somewhat delayed in its appearance in order that its contents might be arranged in conformity with the standard set by the Eighth Decennial Revision of the U. S. Pharmacopœia.

It is trustworthy and up-to-date in every detail, and the wide range of accurate knowledge of its authors is reflected in every page.

Dr. Culbreth's work is noteworthy, not only because of its encyclopedic scope but also on account of the great detail he has bestowed upon the minute details.

---

DISEASES OF METABOLISM AND OF THE BLOOD—ANIMAL PARASITES, TOXICOLOGY. Edited by Richard C. Cabot, M.D., Instructor in Clinical Medicine at the Medical School of Harvard University. D. Appleton & Co., New York.

This is the second volume in a series entitled "Modern Clinical Medicine," the first of which was Infectious Diseases, edited by J. C. Wilson, Professor of Medicine at the Jefferson Medical College, of Philadelphia.

The work may be regarded as setting forth the most advanced teaching in medicine. It is an epitome of the latest researches in a field hitherto regarded as obscure and almost unexplored. The work is unexcelled and should have a place in the library of every physician. The busy practitioner both in medical centers and in remote hamlets may here find his inspiration and guide. One of the distinguishing features of the work which renders it especially valuable to the practitioner, is the very full discussion of treatment, embracing full diet-lists as well as all the modern aids, such as organotherapy, medical gymnastics, massage, hydrotherapy and electro-therapeutics, without subordinating actual drug treatment.

---

CUSHNY'S PHARMACOLOGY. A Text-book of Pharmacology and Therapeutics: The Action of Drugs in Health and Disease. By Arthur R. Cushny, M.A., M.D., Aberd., Professor of Pharmacology in the University College, London; formerly Professor of Materia Medica and Therapeutics in the University of Michigan. In one handsome octavo volume of 752 pages, with 52 illustrations. Cloth, $3.75 net. Lea Brothers & Co., Publishers, Philadelphia and New York, 1906.

On the publication of its first edition Cushny's Pharmacology was at once accepted as the standard authority in its field, a position which has been strengthened by each succeeding edition.

The author is a diligent, painstaking investigator, who enters personally into his subject, and the knowledge he imparts is largely first hand. His work was the first earnest attempt to cut loose from the dogmatical and impirical methods so long current, and to furnish a scientific treatise on Therapeutics based upon the exact laboratory findings and physiological action of drugs. Dr. Cushny's work gives all that is *actually known* to date and the best of what is generally believed regarding the action and value of drugs.

---

LECTURES ON TROPICAL DISEASES. Delivered at the Cooper Medical College, San Francisco, August, 1905. By Sir Patrick Manson, K.C., M.G., M.D., LL.D., (Aber). F. R. C. P. (Lond.), etc. Price $2.50. W. T. Keener & Co., Chicago.

The present time is a particularly opportune one for the appearance of these lectures on Tropical Diseases, while we are just beginning the construction of the Panama Canal. Equally as appropriate for the delivery of the lectures upon this theme was Sir Patrick Manson. His thorough familiarity with the subject, and his clear, interesting way of dealing with it, enable us to commend very highly this book, and especially to those who contemplate a trip to the tropics. Sir Patrick Manson urges the folly of sending young men fresh from medical colleges to some tropical region to treat diseases they know nothing of.

---

WILLIAMS ON FOOD. Food and Diet in Health and Disease. A Manual for Practitioners of Medicine, Students, Nurses and the Lay Reader. By Robert F. Williams, Professor of Principles and Practice of Medicine in the Medical College of Virginia, Richmond. In one handsome 12mo volume of 392 pages. Cloth, net, $2.00. Lea Brothers & Co., Publishers, Philadelphia and New York, 1906.

Being practical, concise, clear and free from technicalities, the above book on foods will undoubtedly prove of distinct value to physicians, nurses and to every family in which sickness has become an unwelcome visitor.

Reference to the tedious scientific investigations by which chemists and physiologists have reached the present day knowledge of diet have been omitted. Results, facts and clear directions are given, and make it a book doctors can recommend to their patients as a guide to the preparation and use of food in sickness and convalescence.

---

KOPLIK ON DISEASES OF CHILDREN. A Treatise on the Diseases of Infancy and Childhood. For Students and Physicians. By Henry Koplik, M.D., Pediatrist to Mt. Sinai Hospital, Ex-President American Pediatric Society, etc., New York. New (2d) Edition. Revised and Enlarged in Text and Illustrations. Octavo, 868 pages, 184 engravings and 33 plates. Cloth, $5.00; Leather, $6.00, net. Lea Brothers & Co., Publishers, Philadelphia and New York, 1905.

The clinical characters of this work, like the first edition, has been preserved throughout, as the author has adhered to his purpose of furnishing a practical guide and text-book for both students and physicians. Merely theoretical and unpractical matters have been carefully avoided, as out of place in a treatise with this design.

A number of new engravings have been added and the text shows an increase of nearly 200 pages. We have every reason to predict for this work the warm welcome from practitioners, teachers and students, with which the first edition was received.

---

FORTY YEARS AN ADVERTISING AGENT. By George Presbury Rowell. Printers' Ink Publishing Co., New York.

This interesting book comprises fifty-two papers that were published in *Printers' Ink*. They publish a valuable contribution to the history of newspapers and advertising in America. "They are full of instruction and profitable suggestions to every man who prints and thinks."

Mr. Rowell's style is terse, humorous and reminiscent.

We commend the book to all who are in any way interested in advertising.

---

### TRYPANOSOMIASIS IN THE ANGLO-EGYPTIAN SUDAN.

Balfour (*Edinburgh Medical Journal*, September, 1905,) calls attention to the following points: The extensive prevalence of trypanosomiasis in the Sudan; the presence in cattle of a small trypanosome which Laveran has declared to be a new species and has named T. nanum; the question as to whether equines or at least mules are liable to a double infection by two different species of trypanosomes, or are the hosts of a T. dimorphum resembling that which affects horses in Senegambia; the great frequency of hemorrhagic ulcerative lesions of the stomach in trypanosomiasis and their significance, also the comparative frequency of intestinal ulceration; the occasional presence of spirilli in these gastric lesions, both in the blood-clot adherent to the ulcers and in the ulcerated surfaces; the action of chrysoidine as a therapeutic agent in trypanosomiasis; the therapeutic action in trypanosomiasis of the blood serum of wild animals (big game) whose habitat is in trypanosome infected areas.

